# Response-code conflict in dual-task interference and its modulation by age

**DOI:** 10.1007/s00426-021-01639-7

**Published:** 2022-02-05

**Authors:** Lya K. Paas Oliveros, Aleks Pieczykolan, Rachel N. Pläschke, Simon B. Eickhoff, Robert Langner

**Affiliations:** 1 Institute of Neuroscience and Medicine (INM-7: Brain and Behaviour), Forschungszentrum Jülich, Jülich, Germany; 2 Institute of Systems Neuroscience, Heinrich Heine University Düsseldorf, Düsseldorf, Germany; 3 Institute of Psychology, University of Würzburg, Würzburg, Germany; 4 Human Technology Center, RWTH Aachen University, Aachen, Germany

## Abstract

Difficulties in performing two tasks at once can arise from several sources and usually increase in advanced age. Tasks with concurrent bimodal (e.g., manual and oculomotor) responding to single stimuli consistently revealed crosstalk between conflicting response codes as a relevant source. However, how this finding translates to unimodal (i.e., manual only) response settings and how it is affected by age remains open. To address this issue, we had young and older adults respond to high- or low-pitched tones with one (single task) or both hands concurrently (dual task). Responses were either compatible or incompatible with the pitch. When responses with the same level of compatibility were combined in dual-task conditions, their response codes were congruent to each other, whereas combining a compatible and an incompatible response created mutually incongruent (i.e., conflicting) response codes, potentially inducing detrimental crosstalk. Across age groups, dual-task costs indeed were overall highest with response-code incongruency. In these trials, compatible responses exhibited higher costs than incompatible ones, even after removing trials with strongly synchronized responses. This underadditive cost asymmetry argues against mutual crosstalk as the sole source of interference and corroborates notions of strategic prioritization of limited processing capacity based on mapping-selection difficulty. As expected, the effects of incongruent response codes were found to be especially deleterious in older adults, supporting assumptions of age-related deficits in multiple-action control at the level of task-shielding. Overall, our results suggest that aging is linked to higher response confusability and less efficient flexibility for capacity sharing in dual-task settings.

**Supplementary Information:**

The online version contains supplementary material available at 10.1007/s00426-021-01639-7.

## Introduction

Performing two tasks at once (i.e., dual-tasking) usually induces costs on performance speed and accuracy, as compared to single-task performance (Koch et al., 2018). Such between-task interference may arise from several sources (Huestegge & Koch, 2010, 2013) and has often been found to be exacerbated with age (Koch et al., 2018; Verhaeghen et al., 2003). Given an increasingly aging society in many parts of the world, it is crucial to investigate the mechanisms behind increasing dual-task interference in advanced age. Although classic dual-task paradigms have been fruitful in identifying how temporal overlap between task processes and the associations between stimuli and responses limit performance, they have been less informative about the mechanisms of interference from output-specific features (Schumacher & Hazeltine, 2016). Thus, we aimed to assess how implementing conflicting versus redundant response rules for both hands affects concurrent responding and how this differs between young and older adults.

It has often been suggested that older adults’ performance may decrease because information is processed more slowly overall. However, a meta-analysis showed that, across studies, older adults demonstrate increased dual-task costs on both speed and accuracy relative to younger ones (Verhaeghen et al., 2003). This over-additive interaction argues against the assumption that age-related dual-task deficits are due to generalized cognitive slowing alone. However, this assumption has not been tested regarding the effects of conflicting response rules in dual-task settings.

### Response-code conflict in dual-tasking

It has long been argued that one mechanism of dual-task interference is *crosstalk* (see Glossary in the Appendix) between input (stimulus) and output (response) features of the two tasks. As such, the features of one task adversely affect the representation of the other task, for instance, by priming a particular but inadequate stimulus interpretation or inducing confusion among *response codes* associated with either task (Hommel, 1998; Navon & Miller, 1987; Tombu & Jolicœur, 2003).

The likelihood of between-task interference through crosstalk is especially increased with high temporal overlap and task similarity, that is, the dimensional overlap between the stimulus–response (S-R) mappings of either task (Hommel, 1998; Navon & Miller, 1987). For instance, Navon and Miller (1987) showed that responses were slower and more error-prone due to the mapping confusability emerging from semantically overlapping stimulus categories in a visual–bimanual paradigm without any stimulus onset asynchrony (SOA) between both tasks. Previous studies have indicated that crosstalk is much facilitated when using a parallel, rather than a serial, processing mode^1^ for coping with two concurrent tasks (Fischer & Plessow, 2015; Lehle & Hübner, 2009). This is especially likely to be the case in settings with short or zero SOAs (Miller et al., 2009). It is frequently assumed that adopting a serial processing mode reflects a strategic choice to minimize between-task confusion and crosstalk (Miller et al., 2009; Navon & Miller, 1987; Tombu & Jolicœur, 2003). However, dual-task crosstalk has remained understudied at the level of responses. Next, we will describe how our implemented approach prevents entering a serial processing mode and thus, maximizes the likelihood of crosstalk.

Employing the *single-stimulus onset paradigm* can shed light on the cognitive mechanisms of crosstalk from outcome or *response-code conflict* in dual-tasking. In this paradigm, a single imperative stimulus calls for two concurrent speeded choice responses (Fagot & Pashler, 1992; Huestegge & Koch, 2009; Pieczykolan & Huestegge, 2014, 2017, 2018). Thus, this paradigm offers the possibility to investigate the processing mechanisms of two-choice reaction tasks^2^ (and their mutual interactions) unconfounded by potential stimulus-related interference, task order, or temporal overlap manipulations used in more traditional dual-task settings.

Using this paradigm with simultaneous oculomotor and manual responses to lateralized auditory stimuli, Huestegge and Koch (2009) demonstrated that dual-task costs significantly increase with *response-code conflict* arising from pairing a compatible oculomotor response with a crossed-hands condition in which the manual response location is incompatible with the location of the tone. A subsequent study found that dual-task costs also increased when concurrent oculomotor and manual responses were in conflict with each other by opposite S-R mappings, i.e., having one response compatible and the other incompatible with the stimulus (Huestegge & Koch, 2010). These findings suggest that response-related crosstalk due to spatial incongruency between response codes (*R-R incongruency*) is a major determinant of dual-task costs, at least under cross-modal dual-response (here, oculomotor) demands (Huestegge & Koch, 2009, 2010; Pieczykolan & Huestegge, 2014). However, because it cannot be excluded that some modality-specific features contributed to the observed asymmetry of the costs for each response modality, it remained unclear whether similar effects would also occur with concurrent responding within the same response modality, for instance, with the left and right hand.

Thus, aiming to shed further light on the open issues related to dual-task interference from response-related crosstalk, we investigated how intra-modal response-code conflict affects dual-task performance and how this differs between young and old adults (Fagot & Pashler, 1992; Huestegge & Koch, 2010; Pieczykolan & Huestegge, 2014; Schumacher & Hazeltine, 2016). We used a single-stimulus onset paradigm (Huestegge & Koch, 2009, 2010; Pieczykolan & Huestegge, 2018) in which we manipulated the compatibility to the stimulus (i.e., *S-R compatibility*) independently for the responses given with either hand. This allowed us to vary the congruency of the response codes used for either hand (i.e., R-R congruency) in dual-response conditions. Thus, using a single output modality (manual only), we created an intra-modal conflict between response codes in R-R incongruent trials, in which participants had to select spatial codes based on mutually incongruent S-R *mapping* rules (i.e., an S-R compatible and an S-R incompatible one, e.g., high pitch—high response for left hand and high pitch—low response for right hand) and apply them to the two concurrently responding hands. Here, *mapping selection* refers to the parallel selection of two concurrent responses to a common stimulus by mapping response codes to two response effectors (hands), as opposed to the notion of an individual serial S-R mapping for each response within the structural bottleneck framework (Huestegge & Koch, 2010).^3^

#### Hypothesis 1

According to Huestegge and Koch’s (2010) model of a central mapping-selection mechanism by which response codes are mapped to different effectors in parallel, we also expected R-R incongruency to elicit substantial crosstalk for concurrent unimodal responses. This crosstalk should result in higher dual-task costs in R-R incongruent conditions, as compared to R-R congruent ones. In particular, response-code-related crosstalk is thought to be elicited by the parallel processing of two conflicting S-R mappings (Huestegge & Koch, 2010). In contrast, with R-R congruency, when there is only one common S-R mapping rule applicable to both hands (compatible *or* incompatible), a single (or conjoint) mapping is thought to be selected, precluding any crosstalk (cf. Fagot & Pashler, 1992).

#### Hypothesis 2

In an earlier single-stimulus onset study (Pieczykolan & Huestegge, 2014), in which an auditory stimulus-induced response-related crosstalk through vocal–oculomotor or vocal–manual responses, the authors showed an interesting cost asymmetry: Independent of response modality, the more difficult S-R incompatible response tended to be prioritized over the easier compatible one, as the larger portion of dual-task costs was transferred to the (easier) S-R compatible response. Based on these and other similar findings with cross-modal response-code conflicts (Huestegge & Koch, 2009, 2010; Pieczykolan & Huestegge, 2014), we also expected the (easier) S-R compatible response to suffer higher dual-task costs in R-R incongruent conditions, while the (more difficult) S-R incompatible response should be prioritized and suffer substantially less.

The mechanism behind such a cost asymmetry under response-code conflict remains to be elucidated, though. A fundamental assumption of parallel processing models of dual-tasking holds that the central processes required for each task’s response selection occurring between stimulus processing and response execution^4^ can run in parallel. Nevertheless, certain costs are registered because central processing capacity is limited. According to the central capacity-sharing model (Navon & Miller, 2002; Tombu & Jolicœur, 2003), mental resources can be shared among two tasks, and the major question concerns their proportional distribution across the tasks. It has been additionally proposed that the allocation of resources is not incidental but the result of a flexible cognitive system that adapts allocation to the particular contextual and task demands (Fischer & Plessow, 2015; Hazeltine et al., 2006; Huestegge et al., 2018; Koch et al., 2018; Lehle & Hübner, 2009; Pieczykolan & Huestegge, 2019). This line of research suggests that resources in dual-task scenarios are allocated strategically according to task difficulty, for instance, towards the a priori more “difficult” response, to overcome crosstalk between tasks. Thus, as discussed above, observing a cost asymmetry in our setting would provide evidence for parallel processing with flexible resource allocation under conditions of response-code conflict.

### Dual-tasking in advanced age

Healthy aging encompasses deterioration in overall processing speed and various cognitive domains, including problems with carrying out two or more cognitive tasks simultaneously (Koch et al., 2018; Verhaeghen, 2011; Verhaeghen et al., 2003). Age-related dual-task deficits have been attributed to difficulties in activating and differentiating two task sets in parallel. For instance, using a cued task-switching paradigm, Mayr (2001) reported increased global set-selection costs in advanced age with ambiguous stimuli and fully overlapping response sets, pointing to task-set similarity as a critical boundary condition for between-task crosstalk to be exacerbated in advanced age (see also Kray & Lindenberger, 2000; Mayr & Liebscher, 2001). This agrees with findings from a dual-task study combining two compensatory tracking tasks, in which dual-task interference in old adults was particularly pronounced with overlap in the response part (Korteling, 1993). Relatedly, Hartley (2001) observed considerably larger age differences in dual-tasking when the response modality was the same, rather than different, in both tasks. That is, when two similar manual responses (vs. a manual and a vocal one) were executed, the chances for response-related crosstalk increased. Allen et al. (2014) also reported age-related interference effects when giving bimanual responses according to two different task sets, but this was tested with two different input modalities (visual and auditory) and varying temporal overlap between tasks.

Furthermore, a recent study (Janczyk et al., 2018) used a simultaneous-onset visuo-motor dual-task to analyze crosstalk effects, combining a letter-type discrimination task with right- or left-hand responses and a concurrent letter-color discrimination task with right- or left-foot responses. This study did not find any significant interaction between age and the (spatial) congruency between lateralized motor response codes, from which it was concluded that both age groups are similarly able to shield Task 1 processing from Task 2 processing. Although at first sight at odds with the difficulties mentioned above in task-set differentiation in advanced age, these findings agree with the notion that such age-related difficulties are predominantly observed in situations with high between-task similarity, which would potentially not apply to the task used in that study (given that it involved responding to two distinct stimulus features using different response modalities, i.e., hand and foot). Thus, its result leaves open the question under which circumstances older adults are particularly susceptible to crosstalk when concurrent responding is based on mutually incongruent response codes. In particular, it has not been examined yet whether between-task crosstalk in advanced age is increased with response-code conflict under conditions of strong conceptual response-set overlap, unconfounded by stimulus- or response-modality features. Thus, an additional aim of this study was to examine how the effects of intra-modal response-code conflict are modulated by age in a dual-task setting that requires concurrent responding to a single stimulus feature (single-stimulus onset paradigm).

#### Hypothesis 3

As mentioned above, aging has been linked to specific deficits in differentiating similar—and therefore easily confusable—task sets (Hartley, 2001; Mayr, 2001). Therefore, older adults should be particularly susceptible to crosstalk between mutually incongruent mapping rules to be activated and applied concurrently, as implemented in our paradigm. However, higher set-selection costs in older participants have previously been observed only with fully overlapping response sets. As parallel dual-tasking (vs. consecutive task-switching) obviates such an overlap, we here examined whether this particular age-related deficit generalizes to a parallel dual-task setting with conceptual response-set overlap (same response modality, same number and relative location of response alternatives), full temporal task overlap, and in the absence of any stimulus interference and response modality effects. We hypothesized that older adults would suffer more strongly under these circumstances than young ones from between-task crosstalk in conditions with response-code conflict.

#### Hypothesis 4

Finally, to examine the potential contribution of generalized slowing to our hypothesized age-related deficits in keeping crosstalk at bay, we implemented the Brinley procedure (Brinley, 1965; Madden et al., 1992), which corrects for global age-related differences in RT across task conditions. If the interaction between experimental factors and age remained statistically significant after this procedure, it would provide strong evidence for a domain- or process-specific age difference, independent of general age-dependent differences in processing speed (Madden et al., 1992). In conclusion, we expected that the detrimental effects of response-code conflict (i.e., higher dual-task costs with R-R incongruency vs. congruency) would be further exacerbated in advanced age, beyond a global age-related deficit in dual-task performance due to generalized slowing.

## Methods

### Participants

An a priori power analysis was conducted using G*Power 3.1 (Faul et al., 2009) for sample size estimation with a *p* = 0.05 and 80% power (two groups with four measurements). Previous pertinent studies reported large effect sizes for both R-R congruency (Huestegge & Koch, 2009; Janczyk et al., 2018; Schumacher et al., 2018) and S-R compatibility (Weis et al., 2015) of ${\eta }_{\mathrm{p}}^{2}$ = 0.44 and ${\eta }_{\mathrm{p}}^{2}$ = 0.59, respectively. Pieczykolan and Huestegge (2014) observed an interaction between S-R compatibility and R-R congruency with an effect size of ${\eta }_{\mathrm{p}}^{2}$ = 0.12. Assuming similar effect sizes for our novel unimodal version of the paradigm would lead to a required minimum total sample size of up to *n* = 12 participants (ANOVA: repeated measures, within factors). However, since reports of age by response-code conflict interactions have been inconclusive (Hartley, 2001; Janczyk et al., 2018), we would consider a small to medium effect of approximately ${\eta }_{\mathrm{p}}^{2}$ = 0.03 as theoretically relevant, yielding a projected minimum total sample size of *n* = 46 (ANOVA: repeated measures, within-between factors).

Based on these considerations and the anticipation of potential drop-outs, we recruited 50 healthy adults (23 young and 27 older adults) to take part in this study. Participants were recruited via advertisements and personal contact and received monetary compensation (20 EUR). All participants reported normal or corrected-to-normal vision and hearing. The majority of participants (*n* = 46) reported right-hand dominance, three participants left-hand dominance, and two did not report any specific hand dominance. All participants gave written informed consent before entering the study. The study protocol was approved by the local ethics committee of the RWTH Aachen University Hospital.

All older participants passed the DemTect screening for dementia (Kessler et al., 2000), indicating that they did not suffer from clinically relevant cognitive impairment (cut-off 13/18 points). Nine participants were excluded from the analysis because of committing ≥ 50% errors in at least one experimental condition. This left us with 20 young (age 18–35 years; *M* = 25.2, SD = 3.7; 13 females) and 21 older adults (age 50–72 years; *M* = 60.0, SD = 7.0; 12 females) for our analyses. In this analysis sample, 37 participants were right-handed, one left-handed, and two did not report any specific hand dominance. Our reduced total sample size of 41 participants still corresponds to a high power of approximately 90%, ensuring that large significant effects were found with at least a power of 80%.

A sensitivity analysis using G*Power 3.1 revealed that our study with a total final sample size of *n* = 41 across two age groups (with four measurements each) would be sensitive to effects of ${\eta }_{\mathrm{p}}^{2}$ = 0.03 with 80% power (at *p* ≤ 0.05) for both within-subject effects and within-/between-subject factors interactions, as well as effects of ${\eta }_{\mathrm{p}}^{2}$ = 0.11 for the between-subject effect.

### Task and procedure

**Fig. 1:**
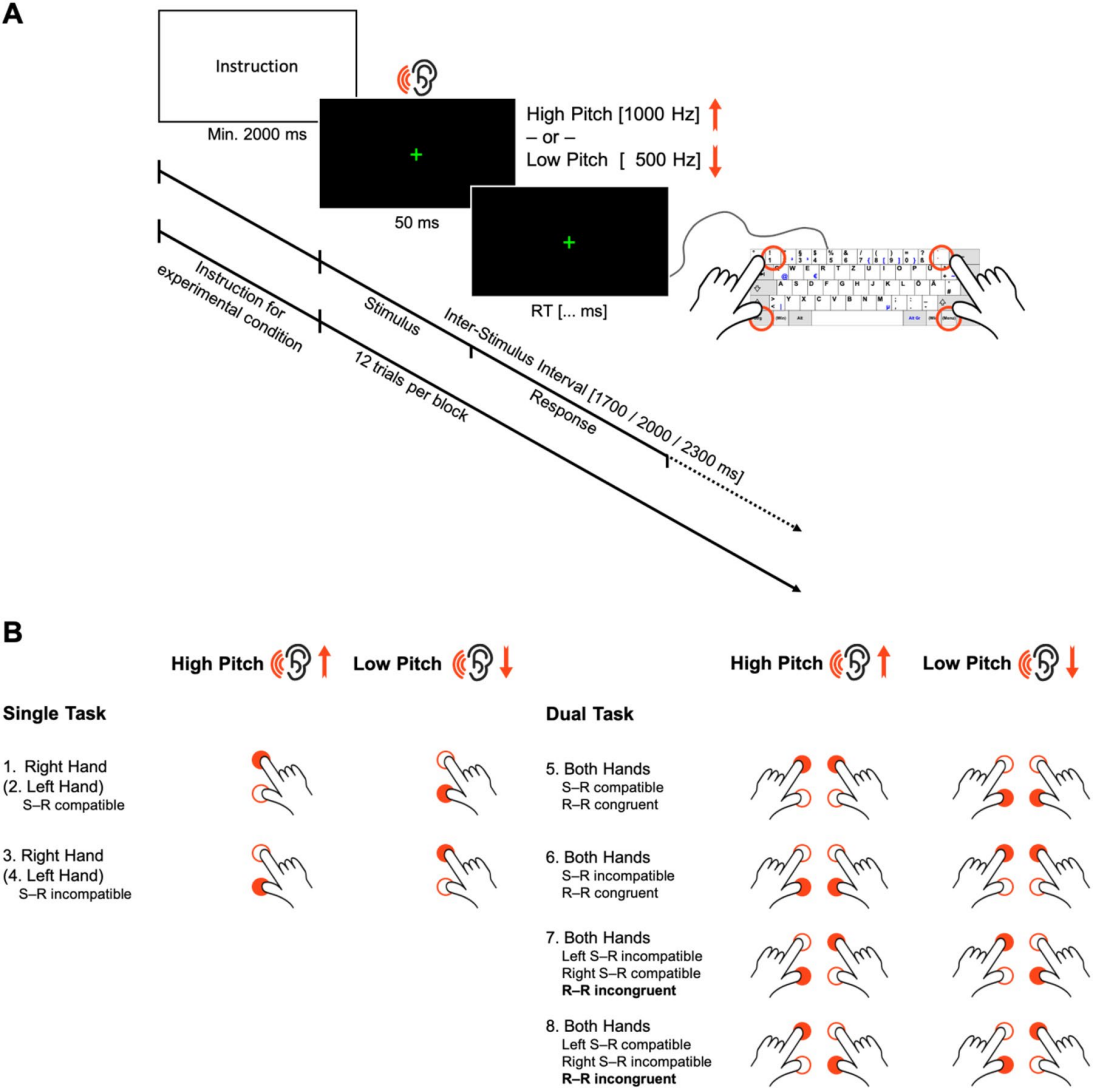
Spatial auditory–manual single-stimulus onset dual-task paradigm. **A** Participants obtained the instruction for the corresponding experimental condition at the beginning of each block. This was followed by 12 experimental trials. In each of them, an imperative low- or high-pitched tone was presented for 50 ms, which had to be responded to with one or both hands concurrently, in a location compatible or incompatible with the auditory stimulus by pressing the higher or lower of vertically positioned response keys (German keyboard layout): “1” for the left and “accent” (first key left to the backspace key) for the right index fingers for “higher” responses, and “left Ctrl” for the left and “right Ctrl” for the right thumbs for “lower” responses. **B** The eight experimental conditions of the dual-task paradigm. *S-R* stimulus–response, *R-R* response–response

The task consisted of presenting high- or low-pitched tones that called for a specific spatial motor response by pressing the higher or lower of two vertically arranged response keys (see Fig. 1). Participants were asked to respond as fast and accurately as possible to the tone pitch with either one (single-task conditions) or both hands concurrently (dual-task conditions). Across blocks of trials, the response mapping was manipulated by varying S-R compatibility: the motor responses were to be executed either in the same or opposite location as implied by the pitch, inducing compatible (e.g., low pitch—low response key) or incompatible (e.g., low pitch—high response key) S-R mappings. This, in turn, allowed us to induce response-code conflicts in dual-task blocks: response codes were either mutually congruent (R-R congruency with both responses being either S-R compatible or S-R incompatible) or incongruent (one response S-R compatible, one S-R incompatible, e.g., combining a low pitch—low button S-R pair with a concurrent low—high one). To this end, the paradigm included eight experimental conditions (see Fig. 1B).

Each experimental block started with a visual instruction of the hand(s) to be used and the S-R mapping(s) to be applied according to the specific experimental condition. Thus, experimental conditions varied between blocks and were presented in pseudo-randomized order to control for potential task-sequence effects. Given the complexity of the paradigm, each participant was presented with only one of the two R-R incongruent conditions (either condition 7 or 8, see Fig. 1B), counterbalanced across the sample. Participants were instructed to maintain fixation on the centrally presented cross throughout the experimental blocks separated by resting breaks (13.5 or 14.0 s, pseudo-randomly varied across blocks). The visual instruction was displayed for a minimum of 2 s and until terminated by the participant via button press, followed by a post-instruction time interval of 1.0 or 1.5 s (varying pseudo-randomly between blocks). Each block comprised 12 trials (stimulus presentation: 50 ms, mean inter-stimulus interval: 2000 ms, pseudo-randomly varying between 1700, 2000, and 2300 ms). For familiarization, participants performed three different blocks (cf. conditions 1, 2, and 5 in Fig. 1B, which were not included in the analyses). Participants completed four blocks of each experimental condition, giving a total of 28 blocks with 48 trials overall per experimental condition for statistical analysis (see Fig. 1).

The current dual-task experiment was part of a more extensive assessment, which additionally comprised several questionnaires, among them the DemTect screening for older adults’ cognitive impairments (Kessler et al., 2000) and other computerized cognitive tasks. The order of task administration was the same for all participants.

### Stimuli and apparatus

A green fixation cross (1°15′0.063″ visual angle) was presented at the center of a black screen. The imperative auditory stimulus was a low- or high-pitched sinus tone (500 or 1000 Hz) presented to both ears via over-ear headphones (Sennheiser HD 201) for 50 ms. Four keys were marked as response buttons on the keyboard (German layout; see Fig. 1A).

Participants sat in front of a 17.3-in. laptop (HP ProBook 470 G4; temporal resolution: 60 Hz; spatial resolution: 1920 × 1080 pixels) at a distance of about 50 cm. A separate keyboard with marked response buttons was placed near the edge of the table, and the laptop was placed directly behind the keyboard. The laptop display was turned open 90 degrees towards the keyboard. The experiment was controlled with Presentation^®^ (Version 18.1, Neurobehavioral Systems, Inc., Berkeley, CA, http://www.neurobs.com) running under Microsoft Windows 7^®^. The experimenter (RP) sat quietly in the room while the participants performed the task.

### Data analysis

For RT analysis, all trials with any incorrect responses were excluded. Anticipatory responses (RT < 150 ms) were also considered as errors. Overly slow correct responses were defined as outliers when their RT was more than three times the standard deviation below or above the individual mean RT of the respective condition and was replaced with the given outlier cut-off. After that, the final RT averages for each experimental condition were calculated. For R-R congruent conditions (requiring either two S-R compatible or two incompatible responses; cf. conditions 5 and 6 in Fig. 1B), we assume a single task representation; thus, RT was averaged across both responses. In contrast, R-R incongruent conditions always contained both a compatible and an incompatible response (e.g., low pitch—low response S-R mapping combined with a concurrent low pitch—high response key), which were analyzed separately. Dual-task speed costs were obtained by computing the difference in mean RT between dual- and single-task conditions of the same S-R compatibility level.

The error rates (ER) were calculated for each condition by adding the amount of omission and commission errors and dividing the sum by the number of total trials per condition. As mentioned above, participants with ER > 50% in at least one condition were excluded from further analysis. Dual-task accuracy costs were computed by subtracting the ER of single-task conditions from analogous dual-task conditions. For R-R incongruent trials, the ERs of the two related single-task conditions (i.e., S-R compatible and S-R incompatible) were subtracted separately, yielding dual-task accuracy costs for either S-R compatibility level under conditions of R-R incongruency.

Statistical analyses were performed with SPSS 24.0 (IBM Corporation, Armonk, NY) and R version 3.6.1 (RStudio, Inc., Boston, MA) and focused on two dependent variables: Dual-task costs on RT and ER. Each dependent variable was submitted to a three-way 2 × 2 × 2 mixed ANOVA. The factorial model included age group (young vs. old adults) as between-subject factor and S-R compatibility (compatible vs. incompatible) and R-R congruency (congruent vs. incongruent) as within-subject factors. Statistical significance level was set to *p* ≤ 0.05 for all analyses.

#### Brinley procedure

To evaluate the generalized slowing hypothesis on dual-task speed costs, we implemented the Brinley procedure (Brinley, 1965) following Madden et al.’s (1992) approach. For this purpose, we regressed the old adults’ group-averaged RT values of all six experimental conditions on the corresponding mean values from younger adults (Brinley, 1965). The resulting linear function (the so-called Brinley function) describes the average increase in RT that can be expected for each observed level of difficulty (i.e., task condition) based on the age difference alone. As such, it captures the generalized (not condition-specific) response slowing related to higher age for a given paradigm.

Madden et al.’s (1992) RT transformation method corrects for the generalized age-related difference in RT across task conditions. This procedure involves transforming the younger adults’ RTs by applying the above linear model of generalized slowing (i.e., the Brinley function) to generate “old-like” RTs in the young group. After that, the statistical analyses are recalculated on the resulting values (i.e., using transformed RT values for the young group and untransformed ones for the old group). In our case, to proceed with the assessment of dual-task speed costs, before the statistical analyses, we computed the difference in mean RT between dual- and single-task conditions of the same S-R compatibility level using the transformed RTs for the young adults and the untransformed ones for the older adults.

## Results

### Dual-task speed costs

**Fig. 2:**
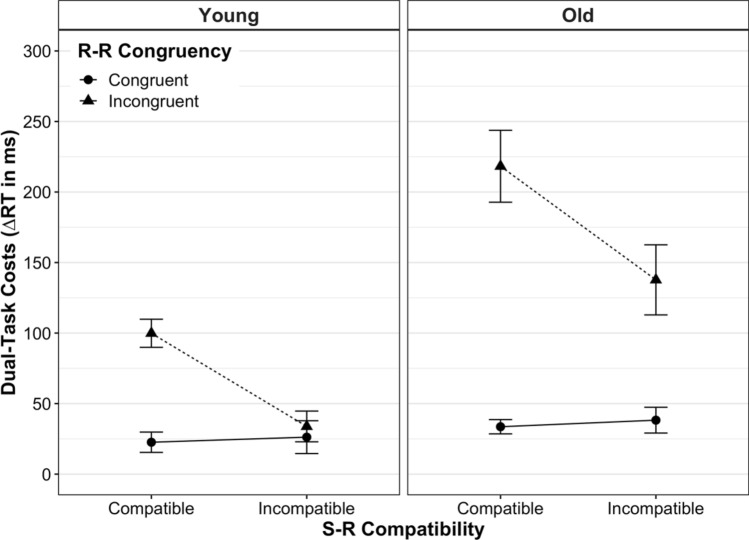
Mean dual-task costs on reaction time (RT) according to age group, stimulus–response (S-R) compatibility and response–response (R-R) congruency. Dual-task costs were obtained through the difference in mean RT between analogous dual- and single-task conditions. Error bars represent the standard error of the mean

Figure 2 shows the mean dual-task costs on RT for young and older adults as a function of S-R compatibility and R-R congruency (see Appendix Table 1 for details on statistics). The absolute RT scores for each experimental condition and age group can be found in Appendix Fig. 6 and Supplementary Table S1.

The mixed three-way ANOVA revealed a significant effect of age, *F*(1, 39) = 18.15, *p* < 0.001*,* ${\eta }_{\mathrm{p}}^{2}$ = 0.32, indicating higher dual-task speed costs in older adults (*M* ± SD: 106.96 ± 113.56 ms) than in young ones (45.62 ± 54.40 ms). There were also significant main effects of S-R compatibility, *F*(1, 39) = 23.33, *p* < 0.001, ${\eta }_{\mathrm{p}}^{2}$ = 0.37, and R-R congruency, *F*(1, 39) = 51.16, *p* < 0.001,   ${\eta }_{\mathrm{p}}^{2}$ = 0.57. However, these main effects were qualified by significant interactions between S-R compatibility and R-R congruency, *F*(1, 39) = 44.80, *p* < 0.001, ${\eta }_{\mathrm{p}}^{2}$ = 0.54, and between R-R congruency and age, *F*(1, 39) = 14.92, *p* < 0.001, ${\eta }_{\mathrm{p}}^{2}$ = 0.28. In particular, the former interaction revealed that the S-R compatibility effect strongly depended on the R-R congruency level: when both response codes were congruent to each other (R-R congruent trials), dual-task speed costs were equivalent between S-R compatible versus incompatible responses in a pairwise comparison (*p* = 0.64). In contrast, when both response codes were incongruent to each other, dual-task speed costs were significantly higher for S-R *compatible* responses (160.50 ± 106.58 ms) than for *incompatible* ones (87.02 ± 101.86 ms, *p* < 0.001). Put differently, although both responses suffered more when being based on mutually incongruent (vs. congruent) location codes, this detrimental impact of R-R incongruency was disproportionately greater on S-R compatible responses than on S-R incompatible ones.

The interaction between R-R congruency and age revealed that the detrimental effect of R-R incongruency on dual-task speed costs was larger in old adults as compared to younger ones. Post-hoc comparisons showed that in R-R incongruent trials, dual-task speed costs were significantly higher for old adults (177.98 ± 121.03 ms) than for younger ones (66.84 ± 56.93 ms, *p* < 0.001), but this was not the case for R-R congruent trials (*p* = 0.18). Finally, we did not find a significant interaction between S-R compatibility and age, *F*(1, 39) = 0.22, *p* = 0.64, ${\eta }_{\mathrm{p}}^{2}$ = 0.01, nor a three-way interaction between S-R compatibility, R-R congruency and age, *F*(1, 39) = 0.45, *p* = 0.51, ${\eta }_{\mathrm{p}}^{2}$ = 0.45.

### Dual-task accuracy costs

**Fig. 3:**
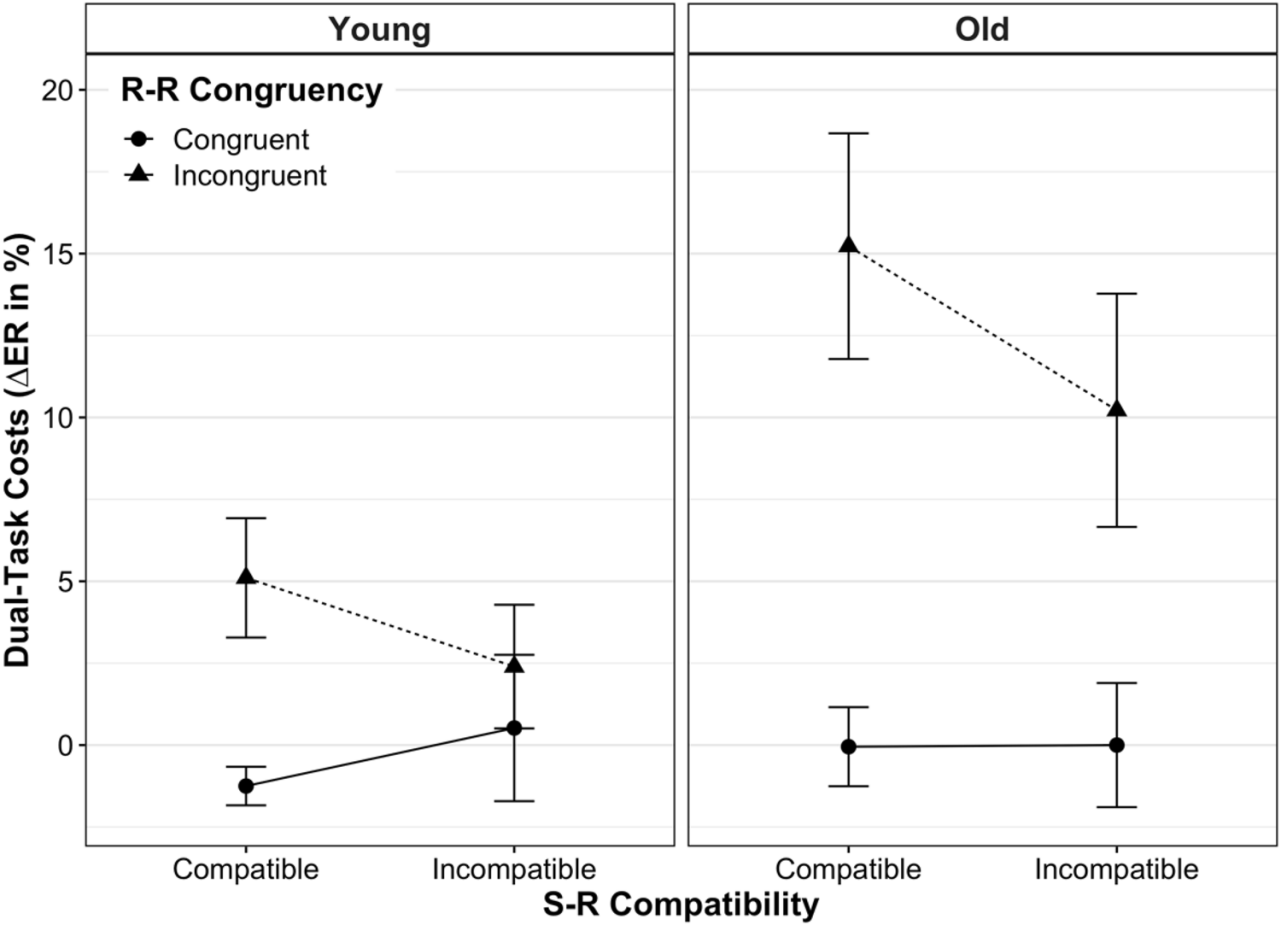
Mean dual-task costs on error rate (ER) according to age group, stimulus–response (S-R) compatibility and response–response (R-R) congruency. Dual-task costs on ER were computed by subtracting the proportion of errors on single-task conditions from their analogous dual-task conditions. Error bars represent the standard error of the mean

Dual-task costs on ER are shown in Fig. 3 for each experimental condition and age group (for absolute ER values, see Figure S1 and Table S1). Overall, accuracy costs followed the pattern of dual-task costs on RT, except that the main effect of S-R compatibility was not significant, *F*(1, 39) = 1.47, *p* = 0.23, ${\eta }_{\mathrm{p}}^{2}$ = 0.04 (for detailed statistics, see Appendix Table 1). In particular, we observed a main effect of age, *F*(1, 39) = 4.38, *p* = 0.04, ${\eta }_{\mathrm{p}}^{2}$ = 0.10, indicating that dual-tasking (vs. single-tasking) induced significantly higher costs on ER in old adults (6.35 ± 13.93%) than in younger ones (1.69 ± 8.02%). The main effect of R-R congruency, *F*(1, 39) = 19.71, *p* < 0.001, ${\eta }_{\mathrm{p}}^{2}$ = 0.34, resulted from higher dual-task costs on ER in R-R incongruent trials (8.35 ± 13.56%) than in congruent ones (− 0.19 ± 7.18%).

The two within-subject experimental factors (i.e., S-R compatibility and R-R congruency) interacted significantly, *F*(1, 39) = 8.91, *p* = 0.005, ${\eta }_{\mathrm{p}}^{2}$ = 0.19, revealing the same relation as observed for dual-task speed costs: in dual-task trials with mutually incongruent response codes, participants showed significantly higher accuracy costs for the S-R *compatible* responses (10.29 ± 13.50%) than for the *incompatible* ones (6.40 ± 13.51%, *p* = 0.003). Conversely, when both response codes were mutually congruent, dual-task accuracy costs did not differ significantly according to the level of S-R compatibility (*p* = 0.59).

Furthermore, the congruency of response codes significantly interacted with age, *F*(1, 39) = 5.17, *p* = 0.03, ${\eta }_{\mathrm{p}}^{2}$ = 0.12. In R-R congruent trials, young and old adults did not show different dual-task costs on ER (*p* = 0.828). However, in R-R incongruent trials, dual-task costs on ER were significantly higher for old adults (12.72 ± 16.05%) than for young ones (3.75 ± 8.30%, *p* = 0.03). In other words, this interaction indicates that old adults’ dual-tasking accuracy costs were affected significantly more by R-R incongruency (vs. congruency) than were those of younger adults. Neither a significant interaction between S-R compatibility and age, *F*(1, 39) = 0.69, *p* = 0.41, ${\eta }_{\mathrm{p}}^{2}$ = 0.02, nor a three-way interaction between S-R compatibility, R-R congruency and age, *F*(1, 39) = 0.03, *p* = 0.88, ${\eta }_{\mathrm{p}}^{2}$ = 0.001, was found.

### Generalized slowing

**Fig. 4:**
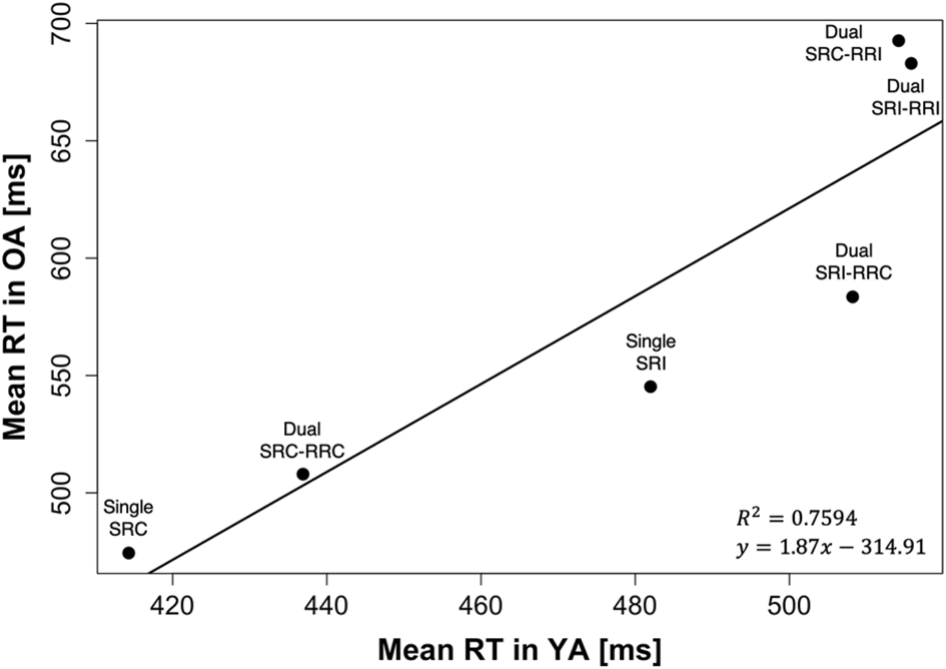
Brinley plot illustrating single- and dual-task mean reaction time (RT) of older adults (OA) as a function of the corresponding mean RT of younger adults (YA) for each experimental condition*.* *SRC* stimulus–response compatible, *SRI* stimulus–response incompatible, *RRC* response–response congruent, *RRI* response–response incongruent

The previous analyses were based on a cognitive model that considers task- or domain-specific processes accounting for the age differences in dual-tasking. However, to address the potential contribution of age-related generalized slowing (Salthouse, 1996) to the observed age-related differences in speeded performance, we examined the relation of mean RT values between young and older adults across experimental conditions (Brinley, 1965; Cerella, 1994). In our case, the old adult’s group-mean RT values shown in Fig. 4 were best represented by the following Brinley function (Brinley, 1965): RT_OA_ = 1.87 × (RT_YA_) − 314.91 (*R*^2^ = 0.76).

The RT transformation yielded average dual-task speed costs of 85.40 ± 101.83 ms for young adults. As the (untransformed) average costs for older adults were 106.95 ± 113.56 ms, the age effect was not significant anymore, *F*(1, 39) = 1.74, *p* = 0.20, ${\eta }_{\mathrm{p}}^{2}$ = 0.04 (see Appendix Table 2 for all statistical results after transformation). The mixed three-way ANOVA again revealed significant main effects of S-R compatibility, *F*(1, 39) = 21.74, *p* < 0.001, ${\eta }_{\mathrm{p}}^{2}$ = 0.36, and R-R congruency, *F*(1, 39) = 47.13, *p* < 0.001, ${\eta }_{\mathrm{p}}^{2}$ = 0.55, qualified by a significant interaction between S-R compatibility and R-R congruency, *F*(1, 39) = 44.64, *p* < 0.001, ${\eta }_{\mathrm{p}}^{2}$ = 0.53, as in all performance scores. However, the previously observed interaction between R-R congruency and age just missed significance, *F*(1, 39) = 3.77, *p* = 0.06, ${\eta }_{\mathrm{p}}^{2}$ = 0.09. Similarly, the post-hoc pairwise comparison also showed only a tendency toward a significant age-related difference in trials with conflicting response codes (125.12 ± 106.57 ms for the young adults and 177.98 ± 121.03 ms for the older adults in R-R incongruent trials; *p* = 0.08). Although just missing conventional significance thresholds and featuring an effect above the smallest effect size of interest (${\eta }_{\mathrm{p}}^{2}$ = 0.03), the results remain inconclusive as to whether the age-related differences observed with untransformed RT data do result from process-specific difficulties with solving response-code conflicts in advanced age or can be explained by generalized slowing after all.

### Supplementary analysis: response grouping

The task design chosen here, involving two concurrent manual choice responses to a common stimulus, is likely to promote synchronized responding, or *response grouping*, on a substantial number of trials (Pashler, 1994). Response grouping refers to a strategy in dual-task settings by which the faster of two reaction processes is slowed by synchronizing its motor execution with that of the second, slower reaction. Although this strategy would not compromise our findings regarding the main outcome of increased dual-task costs with incongruent response codes and the enhancement of this effect in advanced age, we implemented the procedure proposed by Miller and Ulrich (2008) to account for possible grouping-based effects. Here, a cumulative frequency distribution (CDF) of inter-response intervals (IRI) is created to establish an IRI cut-off value of response-grouped trials in dual-task conditions (see Appendix for details on the procedure). The assumption was that if the observed effects remained intact after removing strongly synchronized responses, it would indicate that, at least on the remaining trials, participants engaged a mechanism different from response grouping to cope with crosstalk under conditions of response-code conflict.

**Fig. 5:**
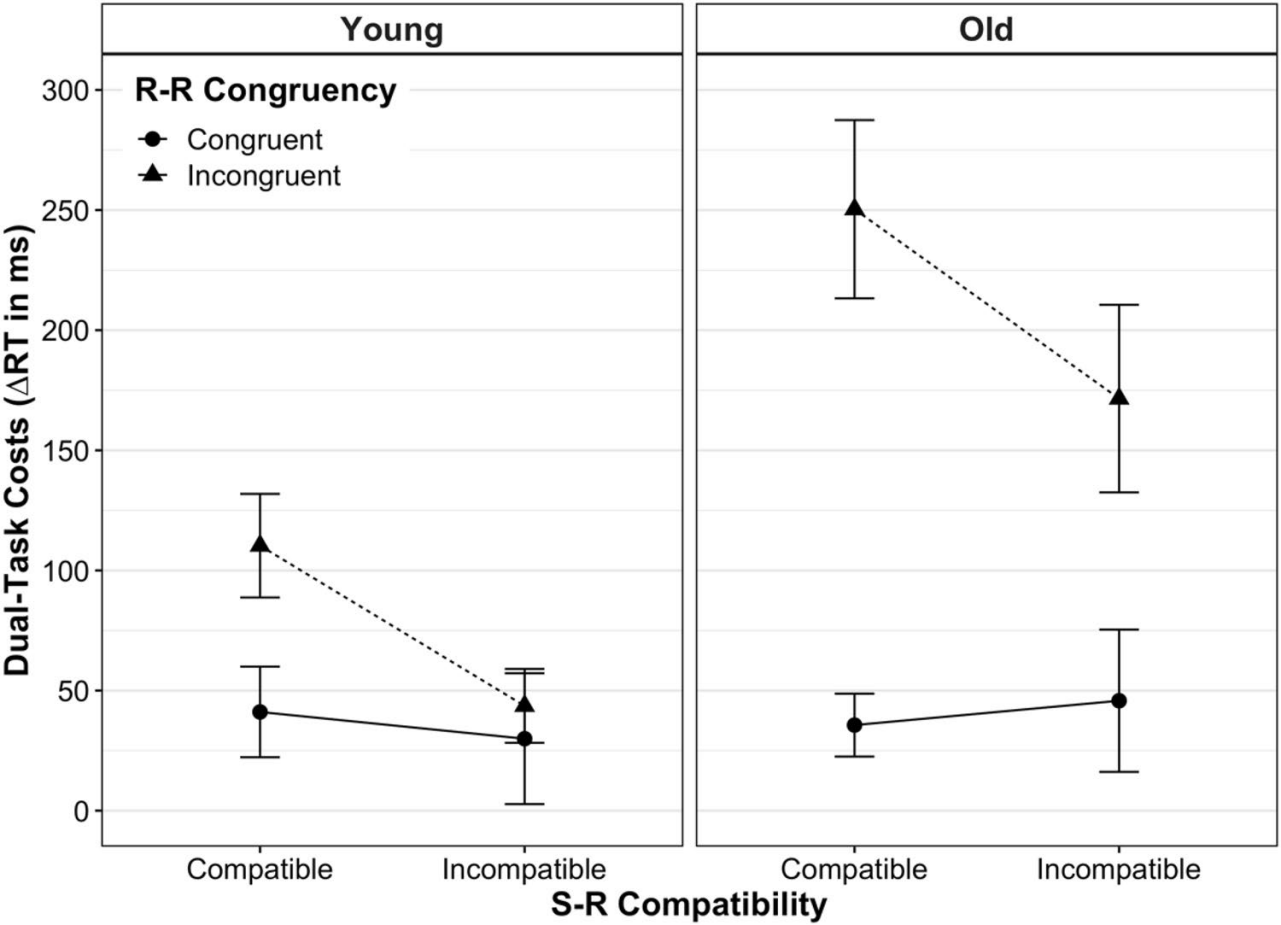
Mean dual-task costs on reaction time (RT) excluding response-grouped trials (cut-off value of IRI ≥ 32 ms) according to age group, stimulus–response (S-R) compatibility and response–response (R-R) congruency. Dual-task costs were obtained through the difference in mean RT between analogous dual- and single-task conditions. Error bars represent the standard error of the mean

We identified an IRI of 32 ms as an appropriate cut-off value for identifying grouped responses in our dual-response setting (see Appendix Fig. 7). After excluding all trials with grouped responses (IRI < 32 ms), we were left with 16 young and 19 older adults because not all participants had enough non-grouped responses in each dual-task condition. When recalculating the dual-task costs on RT in this sample and repeating the mixed three-way ANOVA (see Fig. 5), we obtained the same pattern of results as before, albeit with smaller effect sizes (see Supplementary Table S2 for the detailed statistical results).

Dual-task speed costs were significantly higher in older adults (*M* ± SD: 125.82 ± 161.70 ms) than in young ones (56.24 ± 88.71 ms). There were also significant main effects on these costs of S-R compatibility and R-R congruency. The interactions observed between S-R compatibility and R-R congruency and between R-R congruency and age also remained significant. In more detail, the S-R compatibility reversal effect with R-R incongruency survived as well: dual-task speed costs did not differ between S-R compatible and incompatible responses with R-R congruency (*p* = 0.99), but when response codes for either hand were incongruent to each other, dual-task costs were significantly higher for S-R *compatible* responses (180.32 ± 99.02 ms) than for *incompatible* ones (107.57 ± 90.44 ms, *p* < 0.001). In addition, the interaction between R-R congruency and age showed that in R-R incongruent trials, dual-task speed costs continued to be significantly higher for old adults (210.94 ± 168.54 ms) than for young ones (76.96 ± 81.10 ms, *p* = 0.004), which was not the case for R-R congruent trials (*p* = 0.80). Finally, we neither found a significant interaction between S-R compatibility and age nor a three-way interaction between S-R compatibility, R-R congruency, and age.

Furthermore, we ran the same ANOVA with dual-task speed costs, including only grouped responses (IRI < 32 ms), leaving 20 young and 20 older adults for analysis. As expected, we obtained the same pattern of effects as with the non-grouped responses (see Figure S3 for details).

## Discussion

With the aim of investigating the mechanisms of dual-task response-code conflict in young and older adults, we implemented an auditory–manual single-stimulus onset paradigm with one versus two concurrent speeded choice responses (Huestegge & Koch, 2009; Pieczykolan & Huestegge, 2018). In this paradigm, we varied the compatibility between the location implied by the high or low pitch of the auditory stimulus and the high or low location of the motor response (S-R compatibility; cf. Rusconi et al., 2006). This allowed us to manipulate the congruency between the response codes guiding the two manual responses in dual-response trials (R-R congruency) and elicit response-code conflict through opposing mapping rules (e.g., high pitch—low response combined with a high response). Previous studies have investigated cross-modal response-code conflict in young adults. Here, we assessed the effect of intra-modal (manual only) response-code conflict across the lifespan compared to dual-response conditions with a single, common mapping rule for both responses but with the same types and numbers of stimuli and responses.

### Crosstalk in dual-tasking with response-code conflict

In both age groups, we observed crosstalk effects in trials with intra-modal response-code conflict, as reflected in higher dual-task costs in R-R incongruent versus congruent conditions. First of all, this pattern indicates that our approach to facilitating the confusability of task sets by juxtaposing two largely overlapping unimodal S-R sets with opposing mapping rules did indeed lead to substantial mutual interference. Thus, maximizing the dimensional overlap between tasks with opposing S-R mapping rules in a unimodal setting (cf. Kornblum et al., 1990; Meyer & Kieras, 1997) compares favorably with previous work on crosstalk induced by cross-modal R-R conflicts (Huestegge & Koch, 2009, 2010; Pieczykolan & Huestegge, 2014). Nevertheless, despite the even higher dimensional overlap in our unimodal setting, relative to previous bimodal ones (using very distinct, less automatically synchronized effectors), the average crosstalk effect on RT was somewhat smaller than in cross-modal studies. This is likely due to the differential impact of crosstalk on dual-task costs of S-R compatible versus incompatible responses, as will be discussed below. Overall, we conclude that crosstalk effects in the context of response-code conflict generalize to situations with unimodal mapping selection (e.g., between hands), as compared to bimodal selection (e.g., between hand and eyes).

As expected, dual-task costs on speed and accuracy were not different between S-R compatible and incompatible responses when response codes were congruent to each other. In other words, the performance difference between single- and two-hand responding was not significantly affected by S-R mapping difficulty as long as only a single mapping rule was to be implemented (R-R congruency). This agrees well with the view that such mutually congruent mappings in settings that require two redundant responses at the same time are dealt with via one conjoint response selection process, rather than two independent ones, the result of which is then deployed to both response effectors for execution (Fagot & Pashler, 1992). As such, any dual-response costs in this situation cannot result from interference between two competing response selections but rather reflect general dual-execution costs (Pieczykolan & Huestegge, 2017, 2018). As our paradigm obviates stimulus-related interference, these costs likely arise from demands for two-hand coordination. Consequently, any manipulation that affects response selection, such as S-R compatibility, would be additive to these dual-execution costs.

When, however, two conflicting S-R mapping rules were to be implemented at the same time (R-R incongruent trials), the picture looked somewhat different: S-R compatible responses were slowed down more than S-R incompatible ones when being paired with the opposite mapping, respectively (see full vs. broken lines in all panels of Figs. 2 and 3). Thus, with conflicting response codes, dual-task costs differed between S-R compatible and incompatible responses, similar to what has been reported for cross-modal R-R conflicts in this paradigm (Huestegge & Koch, 2009, 2010; Pieczykolan & Huestegge, 2014). As alluded to above, this situation is markedly different in that it implicates that two different task representations, each associated with a different hand, are represented in parallel. Along the same lines, a recent study using a single visual compound stimulus to trigger uni- or bimanual choice reactions showed that bimanual performance was significantly slowed when the condition implied independent mapping selections, as compared to bimanual conditions with the selection of a single mapping (termed "single task representation"; Schumacher et al., 2018). Although the authors could not pinpoint the mechanism behind this interference resulting from representing the situation as two tasks (vs. only one), effects located at the perceptual or motor level can be excluded.

At any rate, if the interference observed in our data, ensuing from having to handle two distinct (and incongruent) S-R mapping rules for either hand simultaneously, would solely result from between-task crosstalk, this interference should be symmetrical. That is, the costs of dual-tasking should be about equivalent for both S-R compatible and incompatible mapping selections because from a rational observer’s perspective, mapping confusion should cut both ways similarly (i.e., impose costs additively). This, evidently, was not the case.

As mentioned in the “Introduction”, such an asymmetry in dual-task costs is also at odds with a simple central capacity sharing model, in which the first task is thought to receive more processing capacity than the second one (e.g., 80%:20%) (Mittelstädt & Miller, 2017; Navon & Miller, 2002; Pieczykolan & Huestegge, 2019; Tombu & Jolicœur, 2003). In our setting, this model would similarly predict symmetrically increased dual-task costs for both S-R compatible and incompatible responses in R-R incongruent trials, as compared to conditions with congruent response codes, or even lower dual-task costs for the (easier) S-R compatible task (Pieczykolan & Huestegge, 2014). Our findings, however, are consistent with an extended, more flexible model of capacity sharing during dual-tasking, which incorporates the notion of a strategic, task-difficulty-based allocation of limited processing capacity. In particular, Pieczykolan and Huestegge (2014) observed an analogous pattern with conflicting cross-modal response codes and suggested that processing resources are allocated strategically according to perceived task difficulty (such as S-R mapping selection difficulty) for shielding tasks against crosstalk in multiple-task scenarios (Huestegge & Koch, 2010; Pieczykolan & Huestegge, 2014). As a consequence, when a response-code conflict is present (R-R incongruency), the (more demanding) S-R incompatible response mapping would be prioritized and allocated more processing resources. From this, it is predicted that the more difficult mapping is shielded better against crosstalk and, therefore, a relatively larger part of dual-task costs gets conferred onto the de-prioritized (easier) S-R compatible response.

Alternatively, task-set shielding may be biased because top–down cognitive control is already involved in the mapping selection for S-R incompatible responses, aiming to solve the incompatibility-induced response-code conflict (see Rusconi et al., 2006). This more controlled mode of task processing, relative to the more automatic processing of an S-R compatible mapping, should induce an attentional bias that may protect the ongoing mapping selection against conflicting external information (i.e., crosstalk), akin to resistance against distractor interference. According to this view, the observed bias in the effectiveness of (sub)task shielding is more a side-effect or natural consequence of an existing bias in attentional resource allocation to one of the (sub)tasks, while in the original proposal, it results from a strategic decision to allocate more resources based on perceived task difficulty or, possibly, processing fluency. Whatever will be shown in future research to be the exact mechanism, we, demonstrating this bias in a unimodal setting, can already exclude effects that may be specific to cross-modal conflicts such as those potentially related to the dominance of oculomotor over manual response selection, by which the control demands of saccades are prioritized over the control of manual responses (see Huestegge & Koch, 2013).

### Response-related dual-task crosstalk in advanced age

The strong interaction between R-R congruency and age showed that older (vs. young) participants suffer from higher dual-task speed and accuracy costs in conditions with response-code conflict (R-R incongruent vs. congruent conditions). This indicates that older adults have specific difficulties in situations where two mutually incongruent response codes need to be selected concurrently from otherwise highly similar task sets, compared to conditions where two redundant response codes (with a common S-R mapping) need to be selected. Since the age-related results’ pattern of dual-task costs was consistent across both speed and accuracy, we can exclude a difference in the speed–accuracy trade-off between age groups as an explanation. Rather, our results agree with Hartley’s (2001) findings of specific age-related dual-task disadvantages when two similar manual responses have to be executed. Accordingly, the larger response-related crosstalk from opposing mapping rules might be related to an increased response-code confusability, which is further exacerbated in advanced age. The notion of response-code confusability maintains that the concurrent mapping of the same spatial stimulus features (or implications) to a set of motor responses according to different mapping rules for each effector leads to mutual confusion and interference among the spatial codes associated with either effector (Huestegge & Koch, 2009, 2013; Pashler, 1994; Pieczykolan & Huestegge, 2018).

This notion and our findings align with research showing that task-set shielding gets compromised with aging when ambiguity arises from stimuli and response specifications (Mayr, 2001; Mayr & Liebscher, 2001). These age-related difficulties in (consecutive) dual-tasking have been attributed to a potential inhibitory deficit affecting the attentional mechanisms for the processing of one task (i.e., impaired task-set shielding), as well as to a potential deficit in scheduling attention across different task channels, which might cause distraction among parallel processing streams (Hein & Schubert, 2004; Mayr, 2001; Mayr & Liebscher, 2001).

Alternatively, older adults may utilize attentional top–down control to allocate additional processing resources when it is not needed such that performance is not improved but eventually harmed overall. This processing mode has been recently termed the over-reliance on central attention in advanced age and denotes the increased voluntary allocation of attention to central processes as a general strategy to compensate cognitive deficits that come with age (Maquestiaux & Ruthruff, 2021). Accordingly, in trials with response-code conflict, older participants could have overapplied top–down attention to one specific task set, leaving the other one unattended, thereby harming their dual-task performance overall and causing disproportionately higher dual-task costs in the unattended task. For example, older adults might have overapplied attentional resources to the S-R compatible (vs. incompatible) response, although it does not necessarily need it, impeding the automatic activation of response codes eased by the mapping compatibility. Alternatively, it is possible that the elderly overapplied attentional resources to the S-R incompatible response, leaving the compatible one without resources to activate and execute its associated response selection efficiently. As we did not observe a clear age-specific difference in how central attention was distributed between the two mutually incongruent task sets, as reflected in the lack of a three-way interaction, it remains for future studies to investigate in which dual-task crosstalk scenarios different strategies are prioritized by which age group.

To assess whether the detrimental age effect was a domain-specific process, or could instead be explained by generalized slowing, we implemented the RT data transformation proposed by Madden et al. (1992). We observed the same pattern of significant effects and interactions with the transformed values as the ones obtained with the untransformed performance scores (dual-task costs on RT and ER), with the following exceptions: trivially, due to the transformation of the young participants’ RT values, the main effect of age was not significant anymore. Further, the previously strong interaction between age and R-R congruency now showed only a tendency towards significance with a small to medium effect size. Thus, the evidence is not fully clear-cut to distinguish between a generalized slowing explanation or a task-dependent process of motor response-code conflict. More research would be required to disentangle the mechanisms underlying older adults’ performance impairments when two mappings, due to contrary S-R mappings, need to be selected in parallel and, therefore, shielded from each other (Mayr, 2001; Mayr & Liebscher, 2001).

All in all, our results show that the control of response-related conflict, such as the spatial congruency between response codes, in highly similar task sets might be a source of age-related interference. This corroborates notions that older adults face increased difficulties in shifting and shielding multiple task sets or response selection mappings, leading to an increased response-code confusability.

### Alternative explanations for asymmetric dual-task costs with response-code conflict

The observed asymmetry of the increase in dual-task costs for S-R compatible versus incompatible responses in trials with response-code conflict is counterintuitive and at odds with classic models of dual-tasking. In the following, we, therefore, discuss some alternative accounts of this finding.

#### Response selection bottleneck

One of the most influential accounts of the costs arising from dual-tasking is the response selection bottleneck model, which maintains that the central response selection stage cannot be dealt with in parallel when performing two tasks at once. Accordingly, response selection needs to be finished in Task A before it can start in Task B (Pashler, 1994). From this perspective, the overall higher dual-task costs in R-R incongruent (vs. congruent) trials and their asymmetry according to S-R compatibility would be explained by assuming a separate response selection process for either response, implemented in a serial fashion.

Drawing from the pertinent literature on the PRP effect, the response selection stage in auditory-manual two-choice tasks, for S-R mappings without incompatibility, can be estimated to take at least about 200 ms in young adults and roughly 100 ms more in older ones (Allen et al., 2014; De Jong, 1995; see also Hein & Schubert, 2004, for comparable estimates from a similar two-choice visual discrimination task). Assuming that the queuing of the subtasks’ response selections is the only (relevant) source of dual-task costs beyond general dual-execution costs (R-R congruent conditions), it follows from the observed asymmetric cost pattern that the S-R incompatible subtask must have entered the response selection bottleneck first in most trials, delaying the S-R compatible response selection. This selection order is actually inconsistent with findings from PRP experiments showing that the less time-consuming (“easy”, e.g., S-R compatible) response selection is preferably processed before the more time-consuming (“difficult”, e.g., S-R incompatible) one (Leonhard et al., 2011; Ruiz Fernández et al., 2011). However, let us for now assume this was true in our setting with full temporal task overlap. In this case, the serial-selection model would predict that S-R incompatible responses suffer almost no additional costs from dual-tasking since their response selection would hardly ever be delayed, whereas S-R compatible responses should be strongly delayed by at least 250 or 350 ms in young or older adults, respectively.^5^ Obviously, our results look different: On the one hand, there is a larger-than-expected delay for S-R incompatible responses, which amounts to 10 or 100 ms in young or older adults, respectively, as obtained by contrasting RTs of S-R incompatible trials between (serially processed) R-R incongruent conditions and (conjointly processed) congruent conditions (see Table S1). On the other hand, there is a much smaller delay than hypothetically predicted for S-R compatible responses (80 and 190 ms in young and old in our study). Together, this suggests that subtask order was different (i.e., S-R compatible responses were selected first) in a subset of trials (for details, refer to the last section of “Details on the analysis of response grouping” in the Appendix).

Based on the observed dual-task costs for S-R incompatible responses (resulting from trials where these responses were not selected first and therefore postponed, according to the bottleneck model) and the hypothetical costs that would be expected if those responses never came to be selected first, the ratio of trials in which S-R incompatible responses indeed were postponed can be estimated to be about 5 or 33% in young or older adults, respectively (ratio out of 10 or 100 ms observed costs vs. 200 or 300 ms theoretical costs in young or old, respectively). Accordingly, the expected delay of the S-R compatible response selection in the other 95 or 66% of trials would amount to about 140 or 240 ms (i.e., 95 or 66% of 250 or 350 ms) in young and older participants, respectively. These numbers are still way above the observed dual-task costs for S-R compatible responses in R-R incongruent trials (80 and 190 ms). This, as well as the atypical subtask order implied by the observed cost asymmetry, makes it very unlikely that a serial response selection strategy, subject to a structural bottleneck, is the mechanism behind the dual-task costs we observed in the condition with conflicting response codes.

#### Response grouping

A phenomenon often observed in dual-task paradigms, especially in conditions with two manual-response tasks presented very close together in time, is that participants adopt a strategy termed *response grouping* (Pashler, 1994; Pashler & Johnston, 1989; Ulrich & Miller, 2008) or synchronized responding (Fagot & Pashler, 1992). Using this strategy, a participant would select the first response but hold it in, waiting until the second response is also ready to be executed, presumably because it is easier to emit two (manual) responses simultaneously than to emit them in rapid succession.

As our paradigm with easy-to-synchronize bimanual responses to a common stimulus was likely to promote the strategy of response grouping (without inviting it explicitly), it might be argued that the higher dual-task costs for S-R compatible (vs. incompatible) responses in R-R incongruent trials reflect this phenomenon. In particular, promptly executing the S-R compatible response after finishing its easy and fast selection could have been stalled by waiting for the more difficult and slower S-R incompatible selection to finish. To examine this possibility, we followed Miller and Ulrich’s (2008) procedure to exclude trials with grouped responses and then repeated the statistical analysis. The assumption here was that if the effects obtained with all trials were still present after excluding grouped responses, it would indicate that participants not only engaged in response grouping but, at least in a subset of trials, suffered from asymmetric crosstalk causing differential dual-task interference. The results support this assumption. Thus, it appears that under conditions of response-code conflict, participants implement two different strategies that vary from trial to trial: response grouping and a (possibly strategically) biased resource allocation, both leading to a pattern in which the easier (S-R compatible) response suffers higher dual-task costs. This variability in strategy across trials is in line with findings by Miller and Ulrich (2008), suggesting that the decision on whether or not to group responses on a given trial is made online. It remains for future research to answer the question for the key factors that influence this decision in settings like ours with full temporal overlap between two R-R incongruent tasks.

### Future outlook and relevance

Our study implemented a very specific task combination, focusing on output-specific crosstalk using a single-stimulus onset paradigm. It would need to be tested how well our findings generalize to other more conventional task settings considering input–output modality compatibility effects. We suggest that future studies should assess the relevance of output-related features that interfere with the ability to perform two tasks simultaneously and the changes across age within the context of a content-dependent central interference model. For example, one could test which kinds of response-code conflict increase or decrease the probability of code confusability by manipulating the modalities of effector systems in combination with different single-input systems and controlling for general effector-based processing prioritizations (see Huestegge & Koch, 2013). Furthermore, given the study’s cross-sectional nature, it would be beneficial to use longitudinal designs to analyze developmental and environmental factors that contribute to the age-related dual-task deficits.

The present results also have several implications for product design targeted at older people. In our times, humans regularly use devices that require the simultaneous use of fingers and hands, such as display-control, medical, or navigation devices. As already pointed out by Proctor et al. (2005), considering the age-related increase in response-code confusability and task-shielding difficulties might reduce errors and improve the usability for older adults, especially so in the context of multitasking. It would be particularly interesting to investigate under which specific intra- and cross-modal dual-task scenarios different strategies within the context of task-shielding and over-reliance on central attention are implemented.

Furthermore, fundamental dual-task crosstalk has been largely ignored in aging-related research, despite its potential relevance for detecting preclinical markers of cognitive decline in advanced age. Studying the interference between simultaneous cognitive and mobility-related processes in older adults has become an emerging research field, especially in the context of gait (Beurskens & Bock, 2012; Brustio et al., 2017; Liebherr et al., 2016; Porciuncula et al., 2016; Smith et al., 2016), postural control (Boisgontier et al., 2013; Stelzel et al., 2017), and upper-arm movement (Toosizadeh et al., 2019). It seems that performing mobility or postural tasks becomes less automatic with age, negatively affecting secondary cognitive tasks. These observations have been attributed to a possible decrease in the ability to allocate and share attentional resources between mobility-/posture-related and cognitive tasks among the elderly (Boisgontier et al., 2013; Brustio et al., 2017). Identifying early changes within clinical settings could possibly predict frailty and disability and allow quick interventions to prevent a speeded disease progression or adverse outcomes (Brustio et al., 2017; Ceïde et al., 2018; Liebherr et al., 2016).

## Conclusions

Altogether, our study demonstrates that dual-task performance is specifically impaired in settings with intra-modal response-code conflict, extending similar findings on cross-modal interference. Intriguingly, S-R incompatible responses exhibited smaller costs than did compatible ones when response codes were incongruent to each other, even after removing trials with strongly synchronized responding. This generalizes the asymmetric dual-task cost distribution noted by Pieczykolan and Huestegge (2014) beyond cross-modal settings. We suggest that response-code conflicts between two concurrently implemented unimodal mapping rules entail outcome-related crosstalk that is flexibly modulated by a strategic prioritization of limited processing capacity and/or biased attention based on mapping selection difficulty as well as response grouping. Together with other recent studies (Hoffmann et al., 2020; Mattes et al., 2020; Schumacher & Hazeltine, 2016), our findings point to relevant mechanisms involved in dual-tasking that are not sufficiently captured by the structural response selection bottleneck model. Furthermore, they support the view that multiple mechanisms, including crosstalk between (sub)tasks and flexible resource allocation, jointly determine performance costs in dual-task settings, at least in those without entirely separate processing streams and with highly similar response sets.

With age, the overall increase of response-related crosstalk in dual-tasking indicates particular age-related deficits in multiple-action control at the level of task-set shielding. The ability to differentiate and concurrently select two simple but spatially incongruent mappings with high dimensional overlap in input and output appears to become a challenge. This might reflect an increasingly suboptimal weighting of attentional resources in advanced age, including a detrimental over-reliance on the top–down allocation of these resources to either task.

### Electronic supplementary material

Below is the link to the electronic supplementary material.

Supplementary file1 (DOCX 466 KB)

## Figures and Tables

**Fig. 6:**
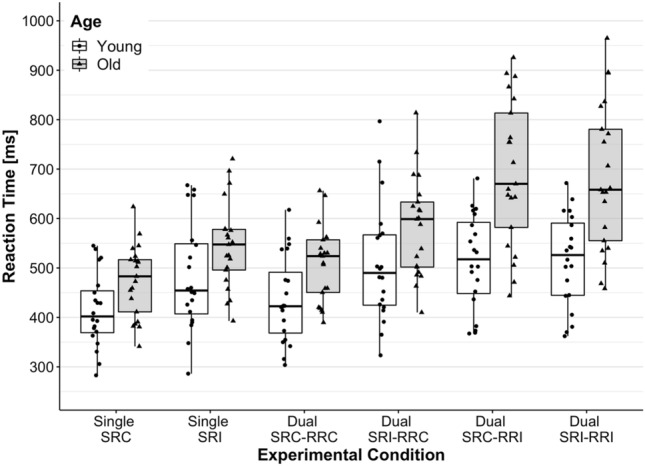
Absolute reaction times as a function of age group and experimental conditions. *SRC* stimulus–response compatible, *SRI* stimulus–response incompatible, *RRC* response–response congruent, *RRI* response–response incongruent

**Fig. 7:**
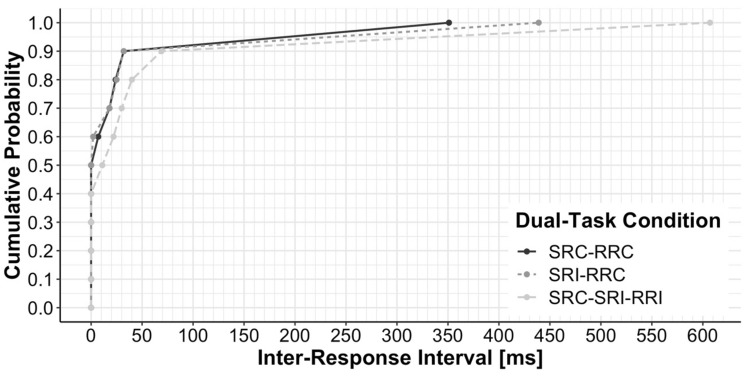
Cumulative frequency distributions of inter-response intervals (IRIs) categorized in deciles across all subjects as a function of dual-task condition. *SRC* stimulus–response compatible, *SRI* stimulus–response incompatible, *RRC* response–response congruent, *RRI* response–response incongruent

## Data Availability

Our participants did not consent to share their data with any third party for reuse; therefore, the conditions of our ethical approval do not permit public archiving of anonymized study data. Thus, data are not generally available to individuals for independent analysis. Data can and will only be released to researchers who agree to collaborate with the principal investigators, e.g., through a formal collaboration agreement. No part of the study procedures and analyses was preregistered in a time-stamped, institutional registry prior to the research being conducted.

## Glossary

### Crosstalk

Non-intentional information transmission between processing streams of different (sub)tasks, which becomes more likely if the tasks share physical features or conceptual dimensions such as overlapping response alternatives (Navon & Miller, 1987).

### Mapping selection

Processing stage in multitasking in which task-relevant response codes are bound to stimulus feature codes in accordance with task instructions (Pieczykolan & Huestegge, 2017).

### Response code

Instructed action for a given effector in response to a stimulus.

### Response-code conflict

Interference between two mutually incongruent response codes to be selected in parallel. In this study, response-code conflict is elicited by the incongruency between two manual response codes resulting from the opposite mapping of tone pitch to response location for either hand.

### Response grouping

Multitasking strategy by which participants select the response for Task 1 and hold it in until the response for Task 2 has also been selected and is ready to be initiated, presumably because it is easier to emit two responses approximately at the same time than emit them in succession (Ulrich & Miller, 2008).

### Response–response congruency

Dimensional correspondence between response codes in dual-task conditions. In this study, response codes were either congruent (same S-R mapping applied to both responses, e.g., high pitch combined with two high button presses) or incongruent to each other (opposite S-R mappings applied concurrently to each response, e.g., high pitch combined with a high response for the left hand and a low response for the right hand).

### Single-stimulus onset paradigm

Paradigm in which a single imperative stimulus calls for two concurrent speeded choice responses (Huestegge & Koch, 2009, 2010; Pieczykolan & Huestegge, 2018).

### Stimulus–response compatibility

Dimensional correspondence between stimulus and response. In this study, the pitch of the auditory imperative stimulus was either compatible with the spatial dimension of the manual response (e.g., high pitch—high response button) or incompatible (e.g., low pitch—high response button).

## Figures and Tables

Figure 6 and Tables 1 and 2 show additional details of our results.

**Table 1:** Results summary for the analyses of variance

	Young (*n* = 20)		Old (*n* = 21)		ANOVA results				
	*M*	*SD*	*M*	*SD*	Effect/interaction	*df*	*F*	*p*	*η*^2^
Dual-task speed costs (ms)									
SRC-RRC	22.60	32.27	33.60	23.21	Age*	1, 39	18.15	< 0.001	0.32
SRI-RRC	26.21	52.05	38.26	41.88	S-R comp*	1, 39	23.33	< 0.001	0.37
SRC-RRI	99.86	44.57	218.26	116.85	R-R congr*	1, 39	51.16	< 0.001	0.57
SRI-RRI	33.81	48.68	137.71	113.88	S-R comp × R-R congr*	1, 39	44.80	< 0.001	0.54
					Age × S-R comp	1, 39	0.22	0.64	0.01
					Age × R-R congr*	1, 39	14.92	< 0.001	0.28
					Age × S-R comp × R-R congr	1, 39	0.45	0.51	0.01
Dual-task costs on error rate (%)									
SRC-RRC	− 1.25	2.63	− 0.05	5.53	Age*	1, 39	4.38	0.04	0.10
SRI-RRC	0.52	9.99	0.00	8.68	S-R comp	1, 39	1.47	0.23	0.04
SRC-RRI	5.10	8.15	15.23	15.78	R-R congr *	1, 39	19.71	< 0.001	0.34
SRI-RRI	2.40	8.44	10.22	16.31	S-R comp × R-R congr*	1, 39	8.91	0.005	0.19
					Age × S-R comp	1, 39	0.68	0.41	0.02
					Age × R-R congr*	1, 39	5.17	0.03	0.12
					Age × S-R comp × R-R congr	1, 39	0.03	0.86	0.001

*M* mean, *SD* standard deviation, *S-R comp* stimulus–response compatibility, *SRC* stimulus–response compatible, *SRI* stimulus–response incompatible, *R-R congr* response–response congruency, *RRC* response–response congruent, *RRI* response–response incongruent

**p* ≤ 0.05

**Table 2:** Statistical results of the analysis of variance for dual-task speed costs after Brinley procedure to assess task-dependent and generalized slowing effects

ANOVA results				
Effect/interaction	*df*	*F*	*p*	*η*^2^
Dual-task speed costs after Brinley procedure				
Age	1, 39	1.74	0.20	0.04
S-R comp*	1, 39	21.74	< 0.001	0.36
R-R congr*	1, 39	47.13	< 0.001	0.55
S-R comp × R-R congr*	1, 39	44.64	< 0.001	0.53
Age × S-R comp	1, 39	0.98	0.33	0.03
Age × R-R congr	1, 39	3.77	0.06	0.09
Age × S-R comp × R-R congr	1, 39	1.96	0.17	0.05

*S-R comp* stimulus–response compatibility, *R-R congr* response–response congruency

^*^*p* ≤ 0.05

## Details on the analysis of response grouping

To establish an IRI cut-off value of response-grouped trials in dual-task conditions, we implemented the procedure proposed by Miller and Ulrich (2008) as follows: we first computed the IRIs by subtracting RT of the first response given from that of the second response. Then, we plotted cumulative frequency distributions of IRIs categorized in deciles as a function of dual-task condition. Following the rationale of Miller and Ulrich (2008), the cognitively simplest dual-task condition was used to determine the motor execution-related threshold. Thus, the IRI cut-off value for response grouping was defined by a marked reduction in the slope of the cumulative density function (CDF) for the S-R compatible, R-R congruent trials. Excluding all trials with IRIs below the cut-off value, we then repeated the statistical analyses previously described.

Figure 7 in the Appendix shows the CDFs of IRIs categorized in deciles as a function of dual-task condition. The CDF of IRIs in both R-R congruent conditions show a narrow distribution with a marked slope reduction at 90% of the trials with an IRI of 32 ms, indicating that this value may be an appropriate cut-off for identifying grouped responses in our dual-response setting^6^(cf. Miller & Ulrich, 2008). The result demonstrates that, as expected, dual-response compounds with only one common S-R mapping rule (R-R congruent conditions) promote synchronization in a large majority of trials. The CDF of trials with two conflicting S-R mapping rules (R-R incongruent condition; see light-grey dashed line in Fig. 7) shows that fewer but still quite many responses were grouped. Approximately 70% of IRIs in R-R incongruent trials were shorter than 32 ms, while longer IRIs spread out over a wide range (32–607 ms), consistent with the idea that these IRIs mainly represent non-grouped responses. Of note, in 43.53% of the trials in R-R incongruent conditions, the concurrent manual responses were given at the same time (51.59% for young and 34.85% for older adults). On the other hand, in 29.12% of the trials, the S-R compatible response was given before the incompatible (26.20% for young and 32.27% for older adults); and in 27.35%, the incompatible was given before the compatible one (22.21% for young and 32.88% for older adults).

## Abbreviations

ANOVA: Analysis of variance
CDF: Cumulative density function
ER: Error rate
IRI: Inter-response interval
PRP: Psychological refractory period
R-R: Response–response
RT: Reaction time
SOA: Stimulus-onset asynchrony
S-R: Stimulus–response

## Footnotes

1 Serial processing models assume that certain parts, or “stages,” of the two tasks need to be processed sequentially. In particular, it is assumed that the resources needed for response selection can only be allocated to a single task at once. Such a response selection bottleneck entails that the response in Task 2 can only be selected after response selection for Task 1 has been completed. In contrasts, parallel processing models argue that the processes required for response selection can run simultaneously for two tasks—but only at a certain cost because central processing capacity is limited (Koch et al., 2018; Pashler, 1994; Pieczykolan & Huestegge, 2019). These are the most commonly used models to explain dual-task interference. However, it is important to mention that they are not mutually exclusive but rather processing modes, among several mechanisms at work, that can be primed depending on the task settings or instructions.

2 The definition of “task” is often not explicit. Most tasks have been described as discrete associations between stimuli and responses, and this has been the fundamental conceptualization of response selection mechanisms (Norman & Shallice, 1986). However, recent representational hierarchy frameworks have proposed that *task files* or *task sets*, defining the associations between stimuli and responses, need to specify more than physical stimulus features and motor parameters, such as contextual information, internal goals, and other relevant task information (Hazeltine & Schumacher, 2016; Schumacher & Hazeltine, 2016). Accordingly, it is necessary to consider the participants’ internal *task representations* that must be learned to perform the task. Thus, response selection interference may emerge from the difficulty and different ways of delimiting and segregating the representations of multiple competing tasks. It is relevant to take all these parameters into consideration to better analyze the possible underlying mechanisms of dual-task interference.

3 Of note, selecting two concurrent responses to a single stimulus according to one common S-R mapping rule (in our study, equivalent to the R-R congruent condition) has been previously termed *conjoint response selection* by Fagot and Pashler (1992), highlighting the notion that such redundant dual-response compounds obviate the typical response selection bottleneck by relying on a single response selection step whose outcome is then applied to both response effectors.

4 Additional stages have been proposed in extension of the traditional information processing model, including response activation (Fischer & Plessow, 2015; Hommel, 1998; Schubert et al., 2008; Watter & Logan, 2006) and response initiation (De Jong, 1993). Furthermore, it has been suggested that dual-task interference can take place at different stages of information processing. However, as our experimental manipulations specifically target the response selection stage, we limit our discussion to the (simplified) three-stage model of stimulus processing, response selection, and response execution.

5 This delay is derived from the above-estimated duration of 200 or 300 ms for the bottleneck stage in young or older adults, respectively, as drawn from the PRP literature plus an estimated S-R incompatibility-related delay of about 50 ms, as gleaned from our single-task data (see Table S1).
