# Supplementary material for: Response-code conflict in dual-task interference and its modulation by age
Source: Psychol Res. 2022 Feb 5;87(1):260–80. doi: 10.1007/s00426-021-01639-7 (PMC9352817; doi:10.1007/s00426-021-01639-7)
Supplement: Supplementary file 1 — Supplementary file1 (DOCX 466 KB) [file 426_2021_1639_MOESM1_ESM.docx]

# Supplementary Information

**Table S1** Absolute Reaction Times and Error Rates for Age Group and Experimental Condition.

|  | **Young (n = 20)** | |  | **Old (n = 21)** | |
| --- | --- | --- | --- | --- | --- |
|  | *M* | *SD* |  | *M* | *SD* |
| *Reaction time (ms)* |  |  |  |  |  |
| Single SRC | 414.31 | 75.12 |  | 474.39 | 72.68 |
| Single SRI | 481.99 | 109.39 |  | 545.25 | 87.32 |
| Dual SRC-RRC | 436.91 | 87.89 |  | 507.98 | 75.94 |
| Dual SRI-RRC | 508.20 | 119.48 |  | 583.50 | 99.97 |
| Dual SRC-RRI | 514.17 | 95.54 |  | 692.65 | 146.75 |
| Dual SRI-RRI | 515.80 | 92.68 |  | 682.96 | 147.88 |
|  |  |  |  |  |  |
| *Error rate (%)* |  |  |  |  |  |
| Single SRC | 3.44 | 2.29 |  | 3.92 | 4.94 |
| Single SRI | 6.15 | 5.71 |  | 8.93 | 9.09 |
| Dual SRC-RRC | 2.19 | 3.13 |  | 3.87 | 6.12 |
| Dual SRI-RRC | 6.67 | 8.54 |  | 8.93 | 9.84 |
| Dual SRC-SRI-RRI | 8.54 | 8.41 |  | 19.15 | 14.65 |

*Note.* *M* = mean; *SD* = standard deviation; SRC = stimulus–response compatible; SRI = stimulus–response incompatible; RRC = response–response congruent; RRI = response–response incongruent.


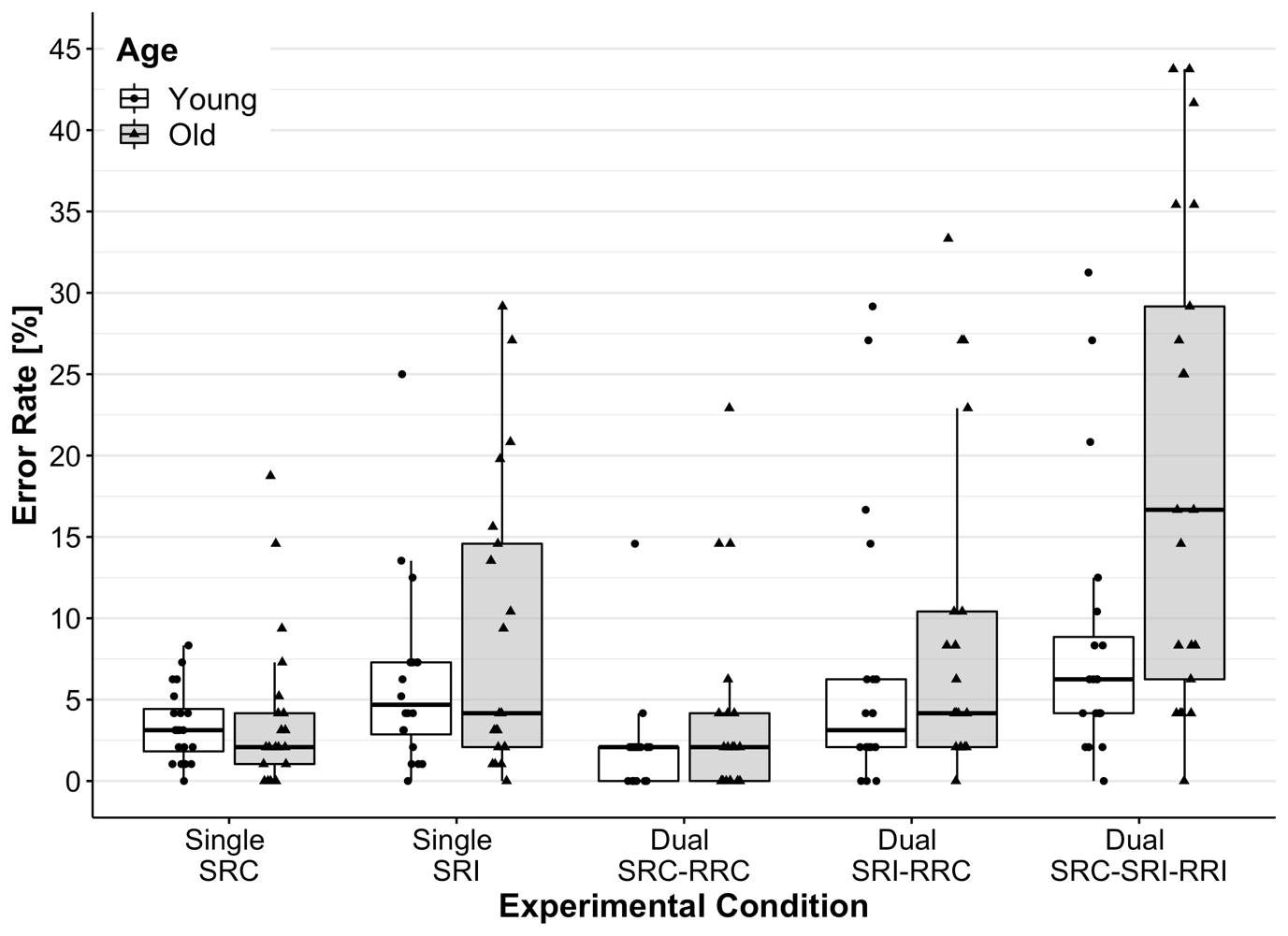


**Fig. S1** Absolute Error Rates as a Function of Age Group and Experimental Conditions. SRC = stimulus–response compatible; SRI = stimulus–response incompatible; RRC = response–response congruent; RRI = response–response incongruent.

**Table S2** Statistical Results of the Analysis of Variance for Dual-Task Speed Costs excluding Response-Grouped Trials (Cut-Off Value of IRI ≥ 32 ms) to Assess Task-Dependent and Generalized Slowing Effects.

|  | **Young (n = 16)** | |  | **Old (n = 19)** | |  | **ANOVA results** |  |  |  |  |
| --- | --- | --- | --- | --- | --- | --- | --- | --- | --- | --- | --- |
|  | *M* | *SD* |  | *M* | *SD* |  | Effect / Interaction | *df* | *F* | *p* | *η^2^* |
| *Dual-task speed costs (ms)*  *excluding Response-Grouped Trials* | | | | | |  |  |  |  |  |  |
| SRC-RRC | 41.10 | 75.48 |  | 35.62 | 57.03 |  | Age * | 1,33 | 9.21 | 0.005 | 0.22 |
| SRI-RRC | 29.95 | 108.93 |  | 45.77 | 129.08 |  | S-R comp * | 1,33 | 6.51 | 0.02 | 0.17 |
| SRC-RRI | 110.31 | 86.25 |  | 250.35 | 161.72 |  | R-R congr * | 1,33 | 18.07 | < 0.001 | 0.35 |
| SRI-RRI | 43.62 | 61.51 |  | 171.52 | 170.16 |  | S-R comp × R-R congr * | 1,33 | 6.43 | 0.02 | 0.16 |
|  |  |  |  |  |  |  | Age × S-R comp | 1,33 | 0.03 | 0.87 | 0.001 |
|  |  |  |  |  |  |  | Age × R-R congr * | 1,33 | 6.69 | 0.01 | 0.17 |
|  |  |  |  |  |  |  | Age × S-R comp × R-R congr | 1,33 | 0.34 | 0.56 | 0.01 |

*Note. M* = mean; *SD* = standard deviation; S-R comp = stimulus–response compatibility; SRC = stimulus–response compatible; SRI = stimulus–response incompatible; R-R congr = response–response congruency; RRC = response–response congruent; RRI = response–response incongruent.

* *p* ≤ 0.05


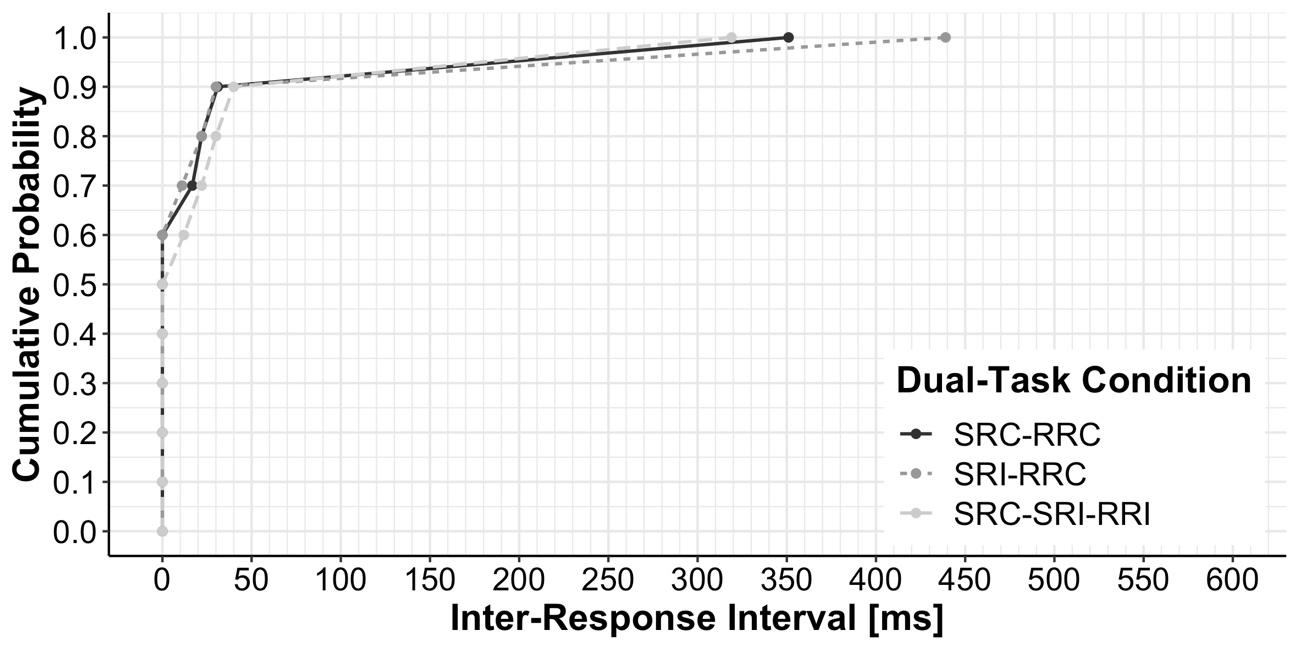


**Fig. S2** Cumulative Frequency Distributions of Inter-Response Intervals (IRI) categorized in Deciles for Young Adults as a Function of Dual-Task Condition. SRC = stimulus–response compatible; SRI = stimulus–response incompatible; RRC = response–response congruent; RRI = response–response incongruent.


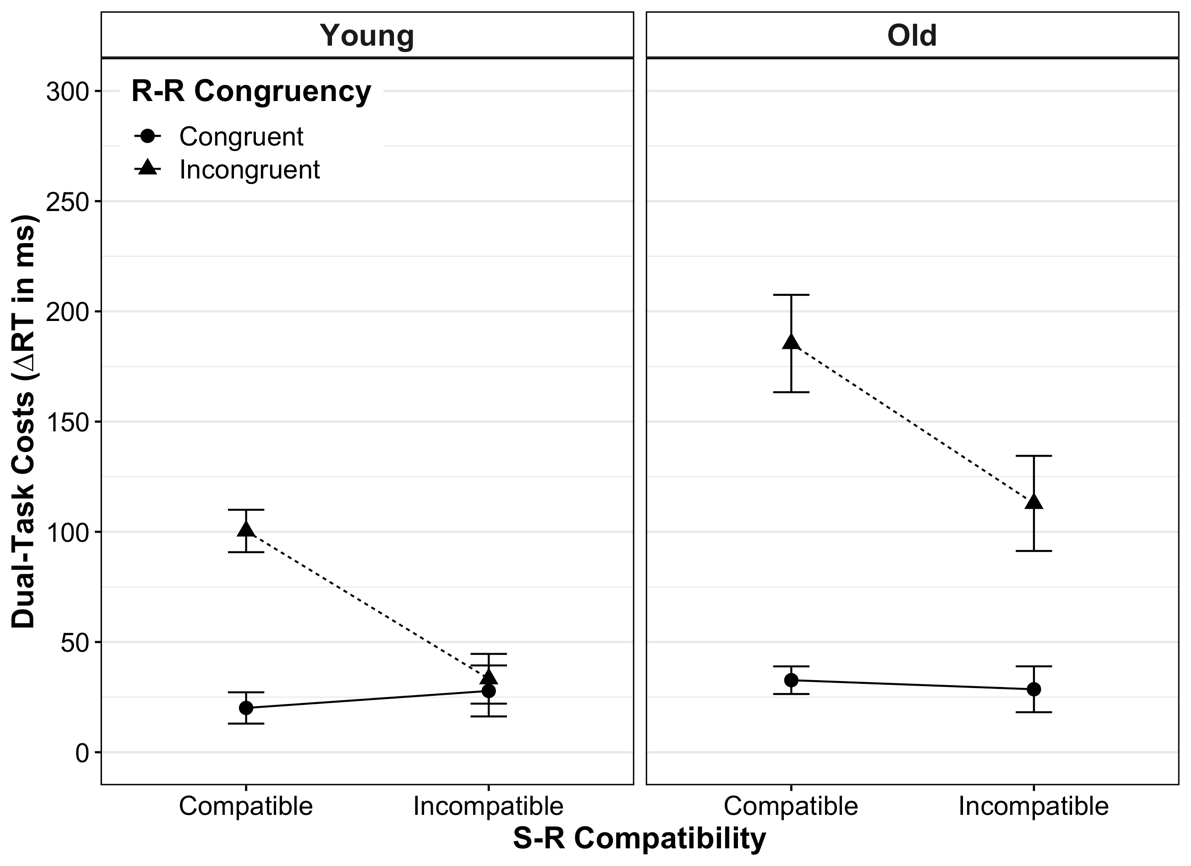


**Fig. S3** Mean Dual-Task Costs on Reaction Time (RT) of Response-Grouped Trials (Cut-Off Value of IRI < 32 ms) according to Age Group, Stimulus–Response (S-R) Compatibility and Response–Response (R-R) Congruency. Dual-task costs were obtained through the difference in mean RT between analogous dual- and single-task conditions. Error bars represent the standard error of the mean.
